# Unveiling Thyrotoxic Periodic Paralysis: A Rare Hyperthyroid Complication

**DOI:** 10.7759/cureus.83280

**Published:** 2025-04-30

**Authors:** Ricardo Cid Puente, Paola D Aguirre Moreno, America M Barrios Muñoz, Ashley M Garcia Luis, Claudia P Contreras Saenz

**Affiliations:** 1 Department of Immunology, Biological Sciences School, Universidad Autónoma de Zacatecas, Zacatecas, MEX; 2 Department of Internal Medicine, Centenario Hospital Miguel Hidalgo, Aguascalientes, MEX

**Keywords:** acute flaccid paralysis, hyperthyrodism, hypokalaemic periodic paralysis, hypokalemic, thyrotoxic hypokalemic periodic paralysis

## Abstract

Thyrotoxic periodic paralysis (TPP) is a rare complication of hyperthyroidism that manifests as recurrent episodes of flaccid paralysis, hypokalemia, and thyrotoxicosis. It can occur in patients with or without a prior diagnosis of hyperthyroidism, and its diagnosis is relatively straightforward once low serum potassium levels and elevated thyroid hormones are evident. However, due to the rarity of the disease, it is seldom the primary diagnosis, and a differential diagnosis must be made with other neurological diseases that resemble the paralysis picture, including Guillain-Barre syndrome. Treatment consists of potassium replacement and management of hyperthyroidism. In this article, we present the clinical case of a previously healthy 24-year-old Latino patient without a history of hyperthyroidism who rapidly developed quadriplegia following intramuscular dexamethasone administration. During his hospital stay, severe hypokalemia and elevated serum thyroid hormone levels were evident, leading to the diagnosis of TPP. Treatment with intravenous potassium and antithyroid medications was initiated, resulting in complete remission of the condition and recovery of mobility in all four limbs. Subsequently, a thyroid ultrasound revealed a thyroid nodule as the cause of thyrotoxicosis. The aim of this work is to raise awareness of a rare disease in the Mexican population, its clinical characteristics, complications, and appropriate treatment and control of recurrent episodes.

## Introduction

Thyrotoxic periodic paralysis (TPP) is part of a group of diseases called periodic paralysis (PP) syndromes, which are characterized by an abnormal shift of potassium ions into muscle cells, causing varying degrees of muscle weakness [1]. It is a rare complication of hyperthyroidism and is predominantly seen in people of Asian descent, with a higher incidence in men. Despite an increase in reported cases within the Caucasian population in the Western world, there are few documented instances within the Latino population [2,3]. The main cause of TPP is Graves' disease (GD), which leads to increased stimulation of the Na-K ATPase pump in the muscle by thyroid hormones, causing a shift of serum potassium into the cell and consequently paralysis [3,4]. Some studies have reported an association with HLA DRw8 and A2BW22 [5]. The clinical presentation is highly characteristic, beginning with a prodromal period of one to three days marked by myalgias and muscle stiffness. This progresses to symmetrical proximal flaccid paralysis, initiating in the lower limbs while sparing the bulbar muscles and cranial nerves, and abolishing osteotendinous reflexes [2,6]. One-third of episodes are triggered by the consumption of large amounts of carbohydrates, alcohol, infections, strenuous exercise, and glucocorticoids, among others. The frequency of crises varies from person to person, but in most cases, they are controlled by achieving a euthyroid state [6,7]. Without treatment, episodes tend to resolve within hours to days [8]. Electrocardiographic alterations are the most common and severe manifestations of TPP and correlate directly with the degree of hypokalemia. On average, serum potassium is below 3.0 mmol/L, and if it is not rapidly identified, it could lead to life-threatening arrhythmias [9-11]. A differential diagnosis must be made with the main causes of paralysis, such as Guillain-Barre syndrome (GBS), myasthenia gravis, and inflammatory myopathies, among others [12]. Treatment consists of potassium replacement and antithyroid medications [13].

## Case presentation

We report the case of a 24-year-old Latino male patient from Aguascalientes, Mexico, without significant family or medical history. He progressively developed mild oppressive pain and weakness in the proximal region of the lower extremities over two days, which worsened while walking. The patient denied gastrointestinal or respiratory infections in the previous days, as well as the use of drugs, medications, or trauma. He sought medical evaluation at a private clinic where he was treated with intramuscular diclofenac and dexamethasone, resulting in partial pain relief but continued weakness. Two hours after medication administration, the weakness gradually progressed to flaccid paralysis of the lower extremities, rapidly evolving to quadriplegia, and he was brought by ambulance to the emergency department (ED) of our hospital.

Upon admission, the patient was conscious, oriented, with vital signs showing a heart rate of 110 beats per minute, respiratory rate of 21 breaths per minute, blood pressure of 122/60 mmHg, SpO2 of 95%, and temperature of 36.0°C. Physical examination confirmed quadriplegia with 0/5 strength in the lower extremities and 1/5 strength in the upper extremities and abolished osteotendinous reflexes. Cranial nerves and sensitivity were spared. Chest and abdominal examinations revealed no abnormalities. Routine laboratory tests in the ED showed severe hypokalemia of 1.9 mmol/L, low serum phosphate of 2.9 mg/dl and low urinary potassium excretion rate of 12.7 mmol/L, with the rest of the laboratories within normal ranges (Table 1).

**Table 1:** Laboratory findings T4: Thyroxine; T3: Triiodothyronine Abnormal laboratory values are indicated in bold

Laboratory tests	Result	Reference range
Hemoglobin (g/dL)	15.3	14.0-16.0
White blood count (1,000/uL)	15.3	4.5-11.0
Platelets (1,000/uL)	301.0	150.0-450.0
Serum creatinine (mg/dL)	0.7	0.6-1.2
Blood urea nitrogen (mg/dL)	16.0	9.0-20.0
Aspartate aminotransferase (UI/L)	26.0	17.0-59.0
Alanine aminotransferase (UI/L)	40.0	0.0-50.0
Lactate dehydrogenase (U/L)	208.0	120.0-246.0
Alkaline phosphatase (U/L)	114.0	38.0-126.0
Serum potassium (mmol/L)	1.9	3.5-5.5
Serum phosphate (mg/dL)	2.9	3.5-4.5
Serum magnesium (mg/dL)	2.9	1.6-2.3
Serum sodium (mmol/L)	141.0	135.0-145.0
Thyroid-stimulating hormone (mIU/mL)	0.001	0.4-4.5
Serum-free T4 (ng/dL)	3.0	0.7-1.4
Serum-free T3 (pg/mL)	6.3	1.5-3.9
Urinary potassium (mmol/L)	12.7	20.0-80.0
Urinary creatinine (mg/dL)	125.0	40.0-120.0

**Figure 1:**
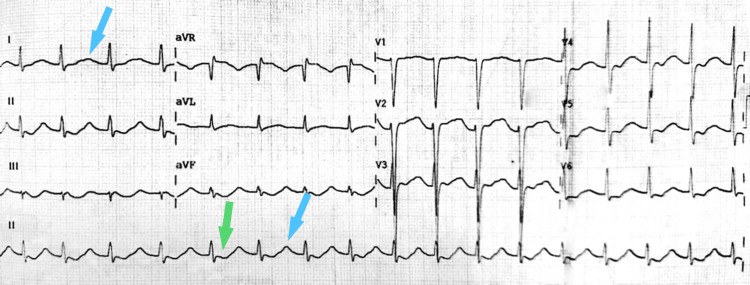
Initial 12-lead electrocardiogram The image shows a 12-lead ECG taken at admission in the ED with hypokalemia-related changes: sinus tachycardia, ST segment depression (green arrow), U waves (blue arrows), and P wave absence. ECG: Electrocardiogram; ED: Emergency department

Due to the potential cardiac complications of hypokalemia, an electrocardiogram was requested, which showed sinus tachycardia, ST segment depression, U wave presence, and P wave absence (Figure 1).

Immediate intravenous potassium replacement was initiated at 40 mEq/h via central line, and a complete laboratory panel was requested, which showed a TSH of 0.001 mUI/mL (0.4-4.5), FT4 of 3.08 ng/dL (0.7-1.48), and FT3 of 6.96 pg/mL (1.58-3.91), values compatible with hyperthyroidism. Consequently, antithyroid treatment with methimazole 5 mg three times a day and propranolol 20 mg once daily was started.

**Figure 2:**
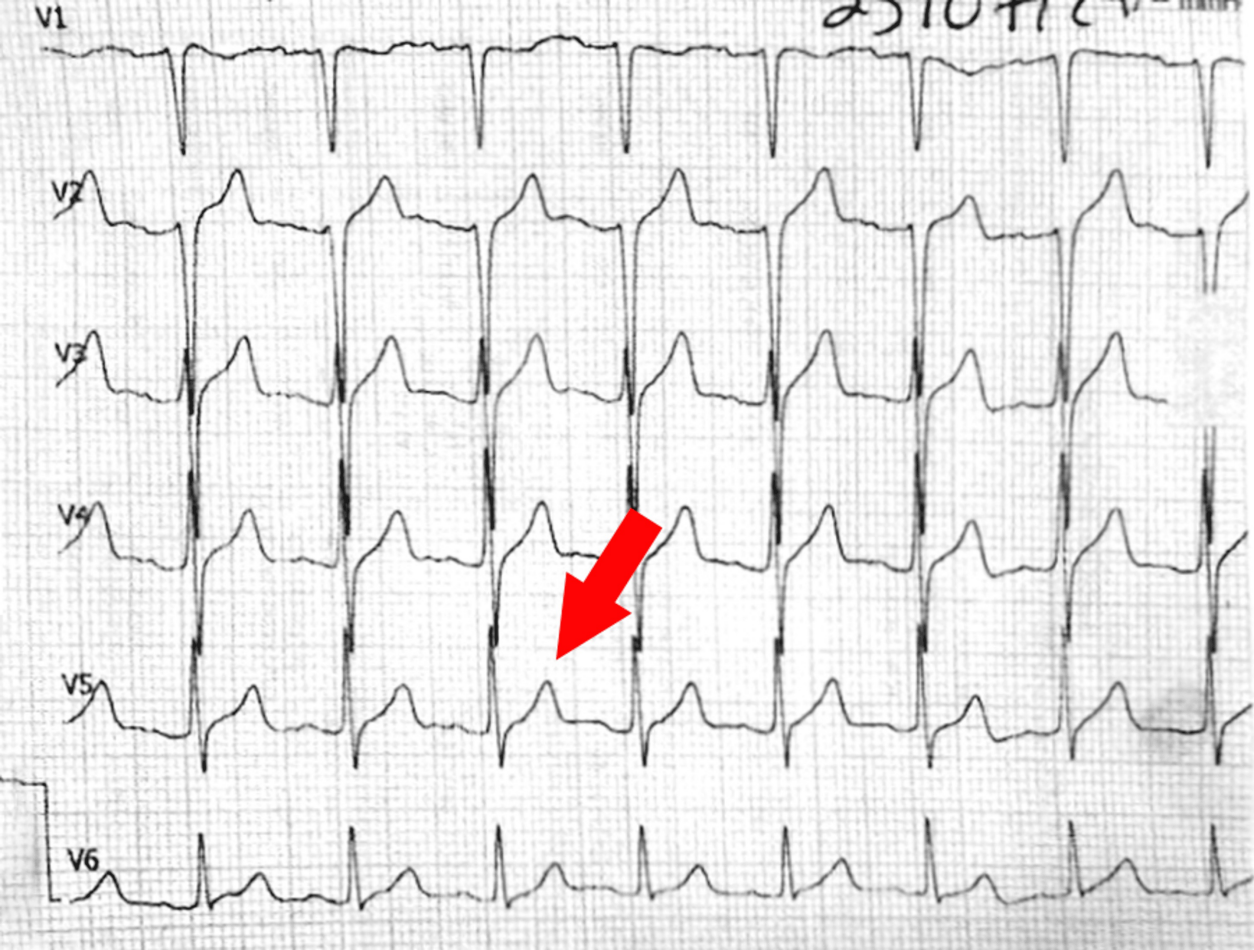
Precordial lead electrocardiogram after potassium replacement The image displays the absence of the U wave and normalization of the ST segment. Peaked T waves (indicated by the red arrow) are observed as a result of excessive potassium replacement.

After the infusion, the patient presented a serum potassium value of 5.7 mmol/L with moderate recovery of limb mobility and a new electrocardiogram showing peaked T waves in precordial leads, probably due to rebound hyperkalemia (Figure 2).

**Figure 3:**
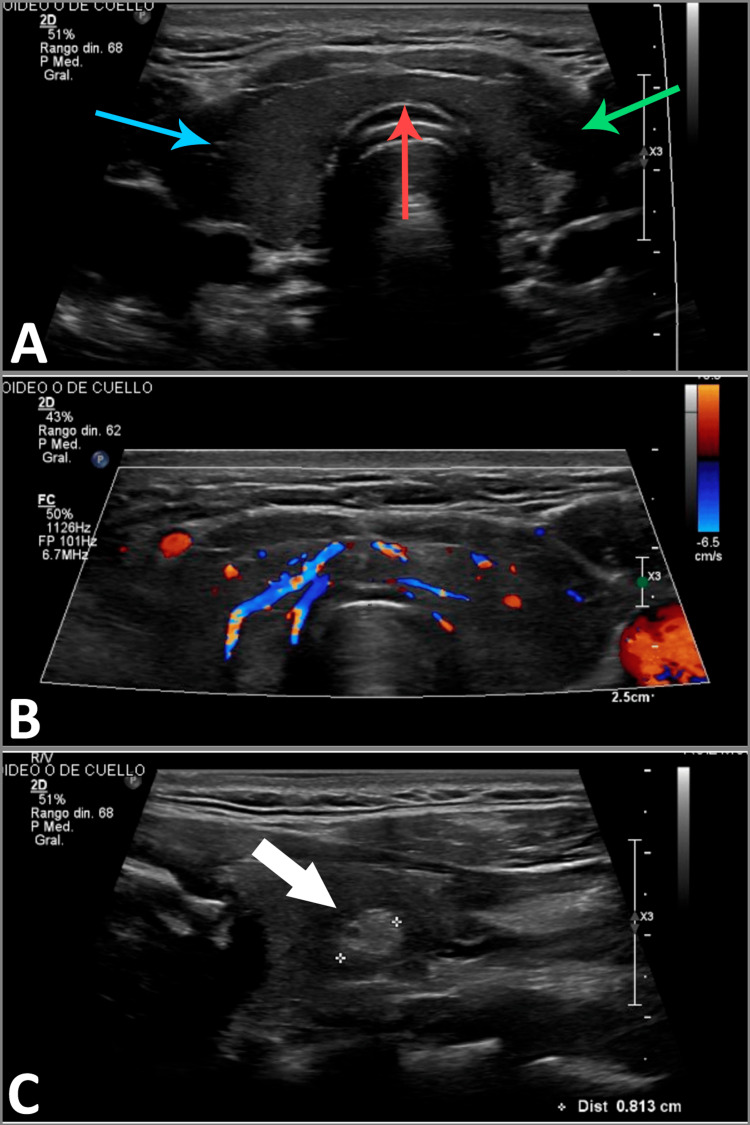
Thyroid gland ultrasound (A) shows thyroid in its normal position and morphology. The right lobe (blue arrow) has a volume of 4.8 mL, the left lobe (green arrow) has a volume of 8.3 mL, and the isthmus (red arrow) has a thickness of 5 mm. The parenchyma is homogeneous. In (B), preserved vascularity is observed with color Doppler. In (C), a hyperechoic image in the left lobe, oval-shaped, circumscribed, avascular, with a diameter of 5x6 mm is observed (Tirads 4a nodule). Tirads: Thyroid Imaging Reporting and Data System

The diagnosis of TPP was made based on the patient's hyperthyroid state, severe hypokalemia, and paralysis that was resolved with potassium replacement. Neck examination revealed no thyroid enlargement, palpable nodules or thyroid bruit, and the patient did not present characteristic signs of hyperthyroidism or GD, so anti-thyrotropin receptors (Trab) and anti-thyroid peroxidase antibodies (anti-TPO) were not determined. The endocrinology service was consulted, and a thyroid Doppler ultrasound was requested, which reported a thyroid nodule in the left lobe, Tirads 4a, measuring 5 x 6 mm with a low probability of malignancy (Figure 3). Due to the lack of resources, thyroid scintigraphy could not be performed.

Twenty-four hours after admission, the patient regained mobility in all four extremities and was discharged with methimazole 5 mg three times a day and propranolol 20 mg once daily. He was referred to the outpatient endocrinology clinic for follow-up.

## Discussion

PP is a group of muscle diseases characterized by dysfunction of transmembrane ion channels, resulting in excessive potassium shift into or out of muscle cells, causing recurrent episodes of muscle weakness and paralysis if untreated. PP is divided into two categories: congenital and acquired. The former is due to an autosomal dominant inheritance pattern and is associated with hypokalemia (familial periodic paralysis (FPP) and Andersen-Tawil syndrome) or hyperkalemia (hyperkalemic periodic paralysis), while the latter, as its name suggests, is an acquired condition that presents with hypokalemia and hyperthyroidism, known as TPP [1]. TPP is an acute complication of hyperthyroidism. It is characterized by recurrent episodes of proximal flaccid paralysis of the extremities in the presence of hypokalemia and thyrotoxicosis. The severity of the condition correlates directly with serum potassium concentration [2].

TPP is extremely rare, primarily affecting the Asian population and occurring between the third and fifth decades of life. Despite hyperthyroidism more frequently affecting women, TPP is more common in men, with a male-to-female ratio of 20:1 and an incidence of 2% in China and Japan. In the United States, an incidence of 0.2% has been reported in the Caucasian population, and few cases have been reported in the Latino population [2,3].

TPP can be triggered by any cause of hyperthyroidism; however, the most frequent cause is GD [3]. TPP is directly developed by thyrotoxicosis without an association with the underlying cause of hyperthyroidism. Despite this, its pathophysiology is not entirely known. The most accepted theory is the stimulation of the Na-K ATPase pump in the muscle by increased thyroid hormones, which promotes the flow of potassium into the cell, leading to a rapid development of hypokalemia. Additionally, adrenergic stimulation by thyroid hormones increases the activity of the Na-K ATPase pump, further increasing the shift of serum potassium into the cell [3,4].

The male predilection could be explained by the increased activity of the Na-K ATPase pump by androgens, and some studies have reported a higher incidence in individuals with HLA DRw8 and A2BW22, specifically in the Japanese and Chinese populations [5]. The clinical presentation begins with a prodrome of myalgias, muscle stiffness, and cramps, usually one to three days prior. It then evolves into transient, episodic, and symmetrical flaccid muscle paralysis, affecting proximal muscles more severely than distal ones. No correlation has been found between the severity of paralysis and thyroid hormone levels. However, during episodes, hypokalemia is almost always present, and the severity of the condition is directly related to serum potassium levels, with muscle strength improving with potassium replacement [2,6]. A precipitating factor can be identified in 34% of patients, mainly the intake of large amounts of carbohydrates, alcohol consumption, infections (respiratory and urinary tract), strenuous exercise, glucocorticoids, hyperinsulinemia, warm weather (summer), and the use of beta-agonist bronchodilators [6]. The frequency of episodes varies from patient to patient and tends to be more common on weekends, which can be associated with dietary changes, alcohol consumption, and increased activity. In most cases, the attacks stop once an euthyroid state is achieved [7].

In patients with TPP, the manifestations of hyperthyroidism can be subtle or obvious and may be present before or coincide with the first episode of TPP [8]. Paralysis can vary from mild weakness to quadriplegia [9]. It presents suddenly and affects the lower extremities more. Osteotendinous reflexes are diminished or absent. Respiratory, ocular, and bulbar muscles are not affected. Sensitivity and higher mental functions are spared. Without treatment, episodes of paralysis tend to resolve spontaneously within hours or up to two days [8].

Rare complications include intestinal pseudo-obstruction, acute hypercapnic respiratory failure, and the most severe, ventricular arrhythmias [2]. Electrocardiographic abnormalities are common and are a consequence of decreased serum potassium. During the acute episode, U waves, QRS widening, QT prolongation, T wave flattening, ST segment depression, atrioventricular blocks, and tachyarrhythmias can be found. The typical triad found in TPP consists of sinus tachycardia, which represents a hyperadrenergic state, QT interval prolongation indicating hypokalemia, and paradoxical PR interval prolongation in sinus tachycardia due to thyrotoxicosis [10].

Thyroid hormones (T3 and T4) are elevated while TSH concentration is decreased. The most frequent laboratory abnormality is hypokalemia, averaging below 3.0 mmol/L, and the rate of potassium excretion in urine is low because hypokalemia is not due to real losses. It is usually accompanied by hypophosphatemia and hypomagnesemia, probably due to intracellular shift, and in some cases, a slight increase in CPK has been described [11].

The differential diagnosis should be made with GBS, FPP, myasthenia gravis, inflammatory myopathies, and transverse myelitis, among others [12].

The treatment of TPP can be divided into two parts: the first consists of the acute treatment of paralysis, and the second in the control of hyperthyroidism to prevent future episodes [2]. The treatment of paralysis consists of potassium replacement; however, special attention should be paid to serum levels since patients with TPP do not have a real potassium deficit but rather a shift of potassium into the cell, so aggressive replacement can result in hyperkalemia [3]. Intravenous potassium administration is preferred, especially in the presence of arrhythmias or severe symptoms, although oral potassium has also been used with the same results. A replacement rate of 10 mEq/h is suggested to avoid rebound hyperkalemia [13]. Non-selective beta-blockers such as propranolol have been shown to improve symptoms by reducing the intracellular shift of potassium and the incidence of new episodes. It is recommended to avoid precipitating factors such as strenuous exercise and high-carbohydrate meals until an euthyroid state is achieved [2]. Finally, antithyroid treatment is the cornerstone to prevent recurrences and should be directed at the underlying pathology causing hyperthyroidism. Treatment options include antithyroid drugs such as methimazole and propylthiouracil, thyroidectomy, and radioactive iodine therapy [13]. Key points for diagnosis of TPP are summarized in Table 2.

**Table 2:** TPP key point summary OTR: Osteotendinous reflexes; TSH: Thyroid-stimulating hormone; FT4: Free thyroxine; FT3: Free triiodothyronine; ECG: Electrocardiogram; TPP: Thyrotoxic periodic paralysis; AV: Atrioventricular; GBS: Guillan-Barre syndrome; FPP: Familial periodic paralysis; IV: Intravenous; TPP: Thyrotoxic periodic paralysis Diagnostic criteria are indicated in bold

	Key points
Age	Third to fifth decade
Gender	More common in men
Clinical presentation	Proximal flaccid paralysis, OTR diminished or absent. Sensitivity, respiratory, ocular, and bulbar muscles are not affected
Triggers	High carbohydrate intake, alcohol, exercise, glucocorticoids, hyperinsulinemia, infections
Laboratory findings	Hypokalemia, low urinary potassium, low TSH, increased FT4 and FT3
ECG findings	Typical triad (sinus tachycardia, QT prolongation and PR prolongation), other common patterns (U waves, QRS widening, T flattening, ST depression, AV blocks and ventricular tachycardia)
Differential diagnosis	GBS, FPP, myasthenia gravis, inflammatory myopathies, transverse myelitis
Treatment	Potassium IV replacement at a rate of 10 mEq/h to avoid rebound hyperkalemia. Beta-blockers. Hyperthyroidism treatment according to its cause. Avoid TPP trigger factors

In the case of our patient, hyperthyroidism was not previously known, and he debuted with TPP, probably triggered by the dexamethasone injection he received. Our case also coincides with the onset of symptoms after a weekend, during which the patient confirmed moderate alcohol consumption upon further questioning. Upon arrival at the emergency department, the initial differential diagnosis was GBS due to the presentation of ascending flaccid paralysis. However, several data points did not align with this diagnosis, such as the sudden onset within hours and the lack of prior infection or vaccination. Nevertheless, once the initial laboratory results were obtained, the diagnosis was evident upon observing the serum potassium value. A key diagnosis aid that correlates with TPP is the presence of low urinary potassium excretion that indicates an intracellular shift rather than potassium losses. In our patient's case, potassium replacement quickly improved the paralysis; however, we observed rebound hyperkalemia that caused electrocardiographic changes, so to avoid complications a less aggressive replacement approach is suggested. Finally, although GD is the main cause of TPP, our patient presented a small thyroid nodule with no signs of malignancy, which has been controlled with antithyroid treatment.

Regarding the limitations of our case, determination of Trab, anti-TPO and thyroid scintigraphy could not be performed due to the lack of equipment to do the tests in our hospital.

## Conclusions

TPP is an extremely rare presentation of flaccid paralysis due to thyrotoxicosis that can mimic several neurological diseases, including GBS. It should be suspected in any young patient with or without a prior diagnosis of hyperthyroidism who presents with muscle weakness and low serum potassium levels. Special attention should be paid to patients with steroid use prior to paralysis as it is a common trigger that often is overlooked. Although primarily seen in people of Asian descent, an increased number of cases have been reported in the Western world and among the Latino population, and it should be considered in the differential diagnosis. The treatment is relatively simple and should be initiated promptly once the diagnosis is confirmed to avoid cardiac arrhythmias. A conservative potassium replacement of no more than 20 mEq/h is advised to prevent hyperkalemia unless severe symptoms/arrhythmias are present. Patients with this condition should be advised to avoid predisposing factors such as high carbohydrate intake, steroids, and alcohol consumption, among others, as well as to adhere to antithyroid medication treatment to prevent recurrence of episodes.
